# Standardizing, harmonizing, and protecting data collection to broaden the impact of COVID-19 research: the rapid acceleration of diagnostics-underserved populations (RADx-UP) initiative

**DOI:** 10.1093/jamia/ocac097

**Published:** 2022-06-09

**Authors:** Gabriel A Carrillo, Michael Cohen-Wolkowiez, Emily M D’Agostino, Keith Marsolo, Lisa M Wruck, Laura Johnson, James Topping, Al Richmond, Giselle Corbie, Warren A Kibbe

**Affiliations:** Department of Pediatrics, Duke University School of Medicine, Durham, North Carolina, USA; Duke Clinical Research Institute, Duke University School of Medicine, Durham, North Carolina, USA; Department of Pediatrics, Duke University School of Medicine, Durham, North Carolina, USA; Duke Clinical Research Institute, Duke University School of Medicine, Durham, North Carolina, USA; Department of Family Medicine and Community Health, Duke University School of Medicine, Durham, North Carolina, USA; Department of Orthopaedic Surgery, Duke University School of Medicine, Durham, North Carolina, USA; Department of Population Health Sciences, Duke University School of Medicine, Durham, North Carolina, USA; Duke Clinical Research Institute, Duke University School of Medicine, Durham, North Carolina, USA; Department of Population Health Sciences, Duke University School of Medicine, Durham, North Carolina, USA; Duke Clinical Research Institute, Duke University School of Medicine, Durham, North Carolina, USA; Department of Biostatistics and Bioinformatics, Duke University School of Medicine, Durham, North Carolina, USA; Duke Clinical Research Institute, Duke University School of Medicine, Durham, North Carolina, USA; Duke Clinical Research Institute, Duke University School of Medicine, Durham, North Carolina, USA; Community-Campus Partnerships for Health, Raleigh, North Carolina, USA; Center for Health Equity Research, University of North Carolina, Chapel Hill, North Carolina, USA; Department of Social Medicine and Department of Medicine, University of North Carolina, Chapel Hill, North Carolina, USA; Department of Internal Medicine, University of North Carolina, Chapel Hill, North Carolina, USA; Department of Biostatistics and Bioinformatics, Duke University School of Medicine, Durham, North Carolina, USA; Duke Cancer Institute, Duke University School of Medicine, Durham, North Carolina, USA

**Keywords:** community research, common data elements, health equity, data privacy, underserved populations

## Abstract

**Objective:**

The Rapid Acceleration of Diagnostics-Underserved Populations (RADx-UP) program is a consortium of community-engaged research projects with the goal of increasing access to Severe Acute Respiratory Syndrome Coronavirus 2 (SARS-CoV-2) tests in underserved populations. To accelerate clinical research, common data elements (CDEs) were selected and refined to standardize data collection and enhance cross-consortium analysis.

**Materials and Methods:**

The RADx-UP consortium began with more than 700 CDEs from the National Institutes of Health (NIH) CDE Repository, Disaster Research Response (DR2) guidelines, and the PHENotypes and eXposures (PhenX) Toolkit. Following a review of initial CDEs, we made selections and further refinements through an iterative process that included live forums, consultations, and surveys completed by the first 69 RADx-UP projects.

**Results:**

Following a multistep CDE development process, we decreased the number of CDEs, modified the question types, and changed the CDE wording. Most research projects were willing to collect and share demographic NIH Tier 1 CDEs, with the top exception reason being a lack of CDE applicability to the project. The NIH RADx-UP Tier 1 CDE with the lowest frequency of collection and sharing was sexual orientation.

**Discussion:**

We engaged a wide range of projects and solicited bidirectional input to create CDEs. These RADx-UP CDEs could serve as the foundation for a patient-centered informatics architecture allowing the integration of disease-specific databases to support hypothesis-driven clinical research in underserved populations.

**Conclusion:**

A community-engaged approach using bidirectional feedback can lead to the better development and implementation of CDEs in underserved populations during public health emergencies.

## BACKGROUND AND SIGNIFICANCE

Standardizing data across multiple research projects may provide greater data utility and clinical insights. By representing data in a standard format, researchers can facilitate better understanding and sharing of data across diverse translational studies.^1^^,^^2^ However, overemphasis on standardizing data in the absence of community input can lead to unintended consequences in clinical research (eg, assumptions that may perpetuate harmful stereotypes, unjustified exclusion of special populations from clinical trials, and poor data generalizability due to low minority enrollment).^3^^,^^4^ For instance, there are rising concerns about “algorithmic bias” in machine-learning applications built on datasets with entrenched patterns of discrimination, limited data resulting from stigma and silence (eg, under-reporting, under-coding) and inappropriate use due to gaps in explainability, trust, and privacy concerns.^5–7^ There are also historical exclusions from clinical trials in special populations such as children,^8^ pregnant women,^9^ patients with chronic medical conditions,^10^ and minorities.^11^^,^^12^ Such exclusionary practices may inadequately capture safety data for new therapeutics, worsen public health emergencies, and produce results that are not generalizable to the broader public.^13^ These outcomes can decrease the representativeness of clinical research and could further harm populations disproportionately impacted by the coronavirus (COVID-19) pandemic.^14^^,^^15^ Thus, there are growing calls for more inclusion of community members from local organizations in the design, implementation, and evaluation of research to better understand cultural differences and community concerns, and help reduce health disparities overall.^16–18^

With the standardizing and sharing of deidentified data in research becoming common practice,^19^ researchers have adopted the use of common data elements (CDEs). CDEs are defined as combinations of standardized questions (variables) paired with a set of responses (values) that are “common” across multiple datasets.^20^ CDEs provide structured, uniform definitions allowing data to be collected, harmonized, and linked across different studies.^21^ In addition, CDEs can facilitate cross-study comparisons, data aggregation, and meta-analyses; simplify training; and promote interoperability between different systems to improve data collection.^22^ Traditionally, CDEs are drafted by subject matter experts, revised after a period of public vetting, and then subsequently released.^23^ These recommended or required CDEs are typically designed for prospective studies that are planned and implemented before the data collection process begins. However, in the ongoing COVID-19 pandemic, a more rapid CDE implementation process was necessary.

The National Institutes of Health (NIH) has developed and deployed CDEs for more than 2 decades and has increasingly endorsed their use in modern clinical trials.^24^ Despite this trend, there exists limited research on CDEs drafted through community partnerships, and even less is known about their implementation in real-world datasets during a global pandemic. Here, we describe the CDE development and implementation process used during the NIH-funded Rapid Acceleration of Diagnostics-Underserved Populations (RADx-UP) program.^25^ RADx-UP is one part of the broader RADx initiative created by the Paycheck Protection Program and Health Care Enhancement Act on April 24, 2020 “to develop, validate, improve, and implement testing and associated technologies” for the COVID-19 pandemic.^26^ Although other RADx initiatives concentrate on development of diagnostic tools, RADx-UP is focused on understanding and addressing COVID-19 impacts on underserved populations across the United States. RADx-UP is a consortium of research projects orchestrated through the RADx-UP Coordination and Data Collection Center (CDCC) led by the Duke Clinical Research Institute at Duke University (Durham, NC) and the Center for Health Equity Research at the University of North Carolina, Chapel Hill, in close partnership with Community-Campus Partnerships for Health (Raleigh, NC).^27^ To date, RADx-UP represents the single largest investment in health disparities research in the history of NIH.^28^^,^^29^ As of early 2022, >100 projects are funded through RADx-UP, serving historically marginalized and medically underserved populations. A list of all current projects can be found on the RADx-UP website (https://radx-up.org/research/projects/list/).

Given the history of inadequate data collection from minority communities,^30^ RADx-UP aims to understand the disproportionate impacts of COVID-19 on these populations. However, efforts to bridge the “data divide” must do so in a responsible and reproducible manner that respects community values, includes data protections, and demonstrates the trustworthiness of research institutions. Such frameworks, if drafted properly, can promote rapid redeployment of CDEs and provide linkable data supporting health officials in prioritizing resources, identifying disparities, and implementing effective short- and long-term interventions. Community-informed CDEs can also provide population-level insights that traditionally drafted CDEs may not capture—these factors include food insecurity, health insurance status, and the social vulnerability index, among other relevant variables.

Although our RADx-UP CDE process had its challenges, and program awardees raised several concerns over data privacy and lack of cultural awareness, we balanced the urgency of the pandemic with the need for scientific integrity, community engagement, and consensus building. The lessons learned through RADx-UP will help further refine the CDEs drafting process, build community relationships with academia, and contribute to a modern blueprint for data collection during and beyond the COVID-19 pandemic.

## MATERIALS AND METHODS

We received Duke Institutional Review Board (IRB) approval for the RADx-UP CDCC (IRB# Pro00106873). As recommended by NIH, we utilized the Disaster Research Response (DR2) CDE development guidelines to establish the RADx-UP CDEs.^31^ Initially, we selected more than 700 CDEs from the NIH CDE repository, DR2 CDE master codebook, and the PHENotypes and eXposures (PhenX) Toolkit. The CDEs were made computable and interoperable according to the PhenX, NLM CDEs, and DR2 guidance document. The DR2 program provides access to a publicly available, curated repository of data collection tools including research protocols, survey instruments, guidance manuals, data dictionaries, and CDEs. We followed the DR2 guideline because its stated objectives aim to catalyze timely and relevant human research in response to public health emergencies. During the pandemic, the DR2 program has supported multiple objectives of the NIH-Wide COVID-19 Strategic Plan. Part of this plan includes drafting of new CDEs to advance knowledge of Severe Acute Respiratory Syndrome Coronavirus 2 (SARS-CoV-2) transmission and population-level impacts.

Building on the NIH CDE repository and DR2 guidelines, we implemented a rapid participatory process for selecting and refining CDEs by engaging NIH and the projects funded in the first phase of the RADx-UP program.

The first phase included 69 RADx-UP projects that were geographically diverse and representative of various academic and community partners from across the United States. The projects were conducting research on a variety of populations (eg, Asian American, Hispanic, Black, or African), in multiple settings (eg, urban, rural, schools, nursing homes), and comprised researchers from various backgrounds and clinical expertise. We conducted a series of meetings and electronic communications from September 2020 through March 2021, and collaboratively analyzed CDE applicability, syntax, and survey responses from these participating RADx-UP projects. In early December 2020, we emailed the 69 RADx-UP projects a link to a REDCap survey requesting feedback on the initial CDE list (see Supplementary Material). We did not have IRB approval specifically for the survey because it was not considered to be human-subjects research. Surveys were sent to each project’s person of contact (their principal investigator or designee), and we asked these project representatives to gather feedback from their teams and submit 1 consolidated response. The survey asked respondents to comment on any questions listed within the 12 categories of CDEs they anticipate being unable to collect. The survey also included free-text fields in each question allowing projects to comment on any anticipated challenges to collecting CDEs, provide any alternative or additional CDEs written from their own perspective, and offer any general feedback on areas such as wording or response options.

The CDCC used this feedback while also considering the 69 RADx-UP projects’ characteristics (eg, populations and settings served by the projects) to consolidate survey responses into the top 5 most reported data concerns. Afterward, the CDCC held a project-wide meeting with the 69 projects in which CDE feedback was reviewed in a question-and-answer session. Following these feedback iterations, the CDCC further refined the NIH RADx-UP CDEs and produced a revised CDE list. This process was designed to help the CDCC further refine and classify the NIH RADx-UP CDEs into required (Tier 1) and recommended (Tier 2) CDEs. In summary, after several rounds of CDE revisions and development steps, including survey feedback from the 69 RADx-UP projects and a subsequent project-wide meeting, the CDCC provided the final list of proposed NIH RADx-UP CDEs to the NIH RADx Executive Committee for approval. Following NIH’s review and approval, we disseminated version 1.0 of the NIH RADx-UP CDEs to all awardees in January 2021. The CDCC provided the Tier 1 and Tier 2 NIH RADx-UP CDE codebook as CSV files, as PDFs with sample forms, and as REDCap dictionaries in English and Spanish to streamline CDE integration into electronic data capture systems. The codebooks and sample forms were made publicly available through the RADx-UP project portal (https://radx-up.org/research/cdes/), the NIH DR2 website (https://tools.niehs.nih.gov/dr2/index), and the NIH National Library of Medicine CDE Browser (https://cde.nlm.nih.gov/home). The provenance for existing CDEs, such as DR2 or PhenX attributions, was included in the codebook. We forwarded the final RADx-UP Informed Consent Data Sharing template document to projects along with the NIH CDE Tracking Form for requesting CDE exceptions and wording modifications (see Supplementary Material). To assist projects with data management, we developed additional training materials in the form of guidance documents, podcasts, newsletters, consultations, and live monthly meetings. These training materials were designed to provide project guidance and recommendations on ways to display CDEs in the forms administered to participants, how to use the codebook, and how to upload the collected CDE data to the CDCC portal.

This cloud-native portal allowed for continuous data submission, data conformance, and quality assurance checks to assess completeness, logical consistency, plausibility, and project compliance with their requested CDE exceptions. We also provided a RADx-UP Data Conformance dashboard to the projects (see Supplementary Figure S1) on which they could assess the following about their own data: number of records uploaded; expected, submitted, conformant, and valid CDEs; completeness; validity; number of data points; and completeness and conformance of data points. We facilitated data quality by providing RADx-UP projects with additional training resources and the data management toolkit references to our website (https://myhome.radx-up.org/cdcc-resources/data-toolkit/). On that site, projects could find links to frequently asked questions, recorded sessions with CDCC leadership discussing CDEs, a data submission guidance document, and tutorial videos to assist projects with common challenges they might encounter while uploading data to the CDCC portal.

Furthermore, we enacted an open-door policy in which projects could contact our CDCC data experts if questions were not covered by the tutorials or materials provided. We leveraged our in-house data experts to facilitate the process and build relationships with cross-consortium stakeholders. The CDCC also sought ways to reduce project workloads prior to transmitting data by ensuring that CDE names directly matched the CDE codebook and were easily retrieved from our website (https://radx-up.org/research/cdes/#files). The CDCC also used a series of core and specialty measures, such as the social vulnerability index, in the RADx-UP CDE PhenX crosswalk to promote the accurate collection of comparable data on social determinants of health across various studies. Lastly, we used Microsoft Power BI to create a RADx-UP CDE Dashboard for better visualization and to foster hypothesis-generating questions of cross-consortium data.

All materials were made available through the “My RADx-UP Home” website (https://myhome.radx-up.org/), and projects could access these materials once registered on the website. We provided data-sharing language templates for the projects to incorporate into their RADx-UP informed consent forms as well as a data transfer agreement template to help facilitate transfers between the awardees and the CDCC (https://radx-up.org/research/cdes/#files) (see also Supplementary Materials). We tracked and monitored implementation of CDEs including any exceptions to collect and share data with the CDCC due to reasons specific to project circumstances.

## RESULTS

Based on the REDCap survey feedback from the first 69 RADx-UP projects and NIH input, we decreased the number of originally proposed CDEs, modified the question types, and changed the CDE wording to increase readability and community acceptance. For instance, we used the PhenX toolkit household relationship CDE, sourced from the General Social Survey (GSS), Household Enumeration Form (HEF) Roster, 2014. The projects refined this list, resulting in the household famgen CDE with questions like “What best describes the people at your home?” Another example involved question on essential workers which originally asked, “Are you a COVID-19 related essential worker or are you in contact with multiple people that are not part of your household?” This CDE raised concern among several projects who felt the wording was difficult to understand and hard to explain. Based on project feedback, this wording was changed to “Are you considered an essential worker? An essential worker is someone who was required to go to work even when stay at home orders were in place.” Several projects also felt that some CDEs (eg, disability status, work email, and SSN) should be optional especially when researching undocumented communities where these CDEs could undermine testing and community member participation. Based on this project feedback, many of these elements were moved to Tier 2 and listed as recommended but not required CDEs.

**Figure 1.**
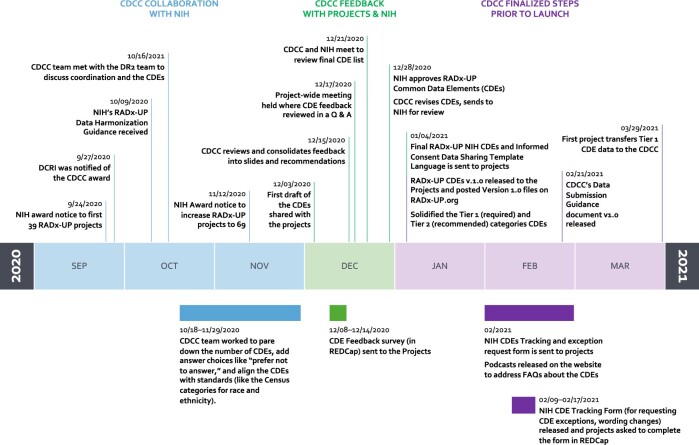
RDX-UP CDE development steps and timeline.

**Figure 2.**
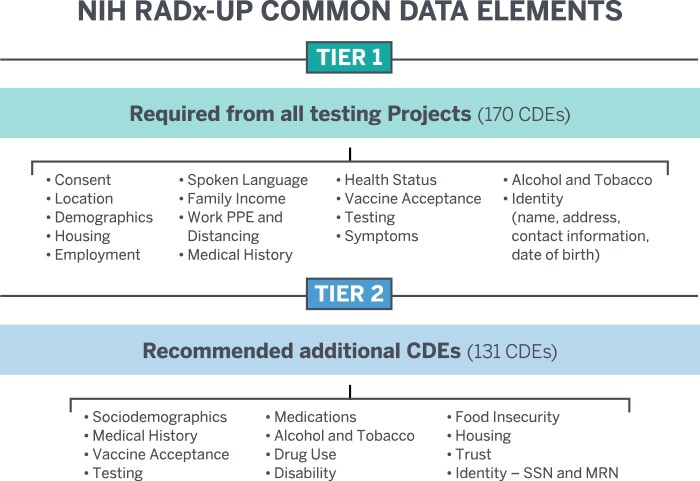
NIH RADx-UP Tier 1 and Tier 2 common data elements (CDEs).

Having selected 170 Tier 1 (required) and 131 Tier 2 (recommended) CDEs, the projects successfully transmitted the first CDEs to the CDCC in March 2021. Figure 1 shows the RADx-UP CDE drafting timeline between September 2020 and March 2021 and the multiple iterations of our CDE development process. As shown in Figure 1, we broke down the timeline into 3 broad categories: (1) early CDCC collaboration with NIH, (2) CDCC feedback with RADx-UP projects and NIH, and (3) CDCC finalized steps prior to launch. Figure 2 highlights the major categories within the 2 NIH CDE Tiers. Table 1 shows the characteristics and demographics of the first 69 RADx-UP projects we surveyed during the CDE drafting process. Table 1 also highlights the populations and setting served by the 69 RADx-UP projects. We carefully considered the REDCap survey responses from these first 69 RADx-UP projects and identified 5 general CDE-related concerns they raised repeatedly: (1) data privacy of underserved and undocumented populations, (2) data burden on participants and projects, (3) data relevance to COVID-19 and RADx-UP research objectives, (4) data sovereignty for tribal nations, and (5) data capture for those projects primarily using electronic health records (EHRs) with no direct patient contact.

**Table 1.** Characteristics and demographics of the 69 RADx-UP projects surveyed

Characteristics	*N* = 69
Populations	
Alaskan Native	2 (3%)
American Indian	19 (28%)
Asian American	23 (33%)
Black or African	54 (78%)
Children	33 (48%)
Hispanic or Latinx	60 (87%)
Native American	0 (0%)
Native Hawaiian	4 (6%)
Older Adults	34 (49%)
Pacific Islander	8 (12%)
Incarcerated People	1 (1%)
Pregnant	18 (26%)
Other	22 (32%)
Settings	
Urban	30 (43%)
Rural	26 (38%)
Community Health	36 (52%)
In-Home	28 (40%)
School	20 (29%)
Nursing Home/LTC	4 (6%)
Prison	1 (1%)
Public Housing	12 (17%)

*Note*: Percentages are rounded to the nearest whole number. Note that a given project can collect in multiple populations and multiple settings.

LTC: Long-Term Care.

Based on these REDCap survey results, data privacy concerns about collecting participants’ protected health information (PHI), Social Security numbers, personal identifiers, and tobacco and alcohol use were the top issue reported by the 69 RADx-UP projects respondents. The second highest concern projects raised was data burden from the volume of CDEs, length, and semantic wording of questions. Third, some projects felt many of the required CDEs asking for all current medications, over-the-counter medicines, past medical history, and disability information were unnecessary and irrelevant to COVID-19 and RADx-UP objectives. Fourth, projects serving tribal nations raised concerns over data sovereignty, control, and legal challenges related to navigating tribal law. Finally, some RADx-UP projects were capturing data directly from EHRs and felt many required CDEs were unnecessary because they had no direct contact with patients.

**Figure 3.**
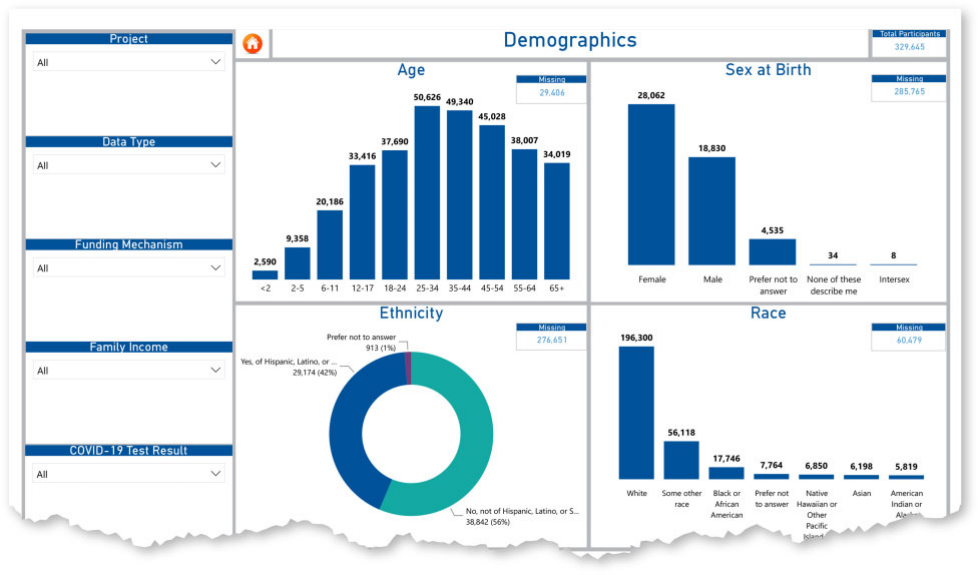
RADx-UP CDE dashboard for demographics.

After considering and incorporating input from these 69 RADx-UP projects’ respondents, we identified the NIH Tier 1 RADx-UP CDEs that consisted of 11 sections with 170 individual elements. Of these, 42 used branching logic, and 28 were yes/no answers. Fourteen CDEs specified information about the identity of the participant (contingent on study design and participant consent). In addition, 131 NIH Tier 2 (recommended) CDEs were selected. Most projects were willing to collect and share demographic NIH Tier 1 CDEs (Table 2), with several requests for exceptions to collecting NIH RADx-UP CDE in sociodemographic (*N* = 35), housing/employment (*N* = 36), medical history (*N* = 25), health status (*N* = 23), vaccine acceptance (*N* = 21), and testing (*N* = 23) as shown in Table 3. Notably, 33–48 out of the first 69 projects did not request an exception for various CDEs presented in Table 3. Outside of pregnancy status, most projects opted not to collect data on gender identity and sexual orientation. As shown in Figure 3, we also created the CDE data dashboard in certain categories to provide projects with visual and interactive representations of CDE data. As of February 2022, 63 of the projects are regularly submitting their data through the CDCC secure cloud portal.

**Table 2.** Number of projects collecting and sharing demographic NIH RADx-UP Tier 1 CDEs (*N* = 69)

	Categories							
Collecting status	Race	Ethnicity	Age	Sex at birth	Gender identity	Pregnancy status	Sexual orientation	Highest education level
Collecting and sharing with the CDCC	66 (96%)	64 (93%)	64 (93%)	60 (87%)	54 (78%)	50 (72%)	45 (65%)	62 (90%)
Collecting but not sharing with the CDCC	2 (3%)	1 (1%)	2 (3%)	2 (3%)	1 (1%)	0 (0%)	0 (0%)	1 (1%)
Not collecting	1 (1%)	4 (6%)	3 (4%)	7 (10%)	14 (20%)	19 (28%)	24 (35%)	6 (9%)

*Note*: Percentages are rounded to the nearest whole number.

**Table 3.** Reasons for exceptions to collecting NIH RADx-UP Tier 1 CDEs

	Categories						
Reasons	Sociodemographic	Housing/employment	Medical history	Health status	Vaccine acceptance	Testing	
	(*N* = 35)	(*N* = 36)	(*N* = 25)	(*N* = 23)	(*N* = 21)	(*N* = 23)	
Not applicable to project/protocol	16 (46%)	16 (44%)	10 (40%)	10 (43%)	10 (48%)	11 (48%)	
Not applicable to study population	15 (43%)	14 (39%)	5 (20%)	5 (22%)	1 (5%)	2 (9%)	
Negative impact on enrollment	14 (40%)	6 (17%)	3 (12%)	4 (17%)	1 (5%)	2 (9%)	
Survey is too lengthy	10 (29%)	11 (31%)	9 (36%)	12 (52%)	9 (43%)	11 (48%)	
Negative impact on community relationship	12 (34%)	6 (17%)	5 (20%)	5 (22%)	2 (10%)	2 (9%)	
Community Advisory Board advice	11 (31%)	11 (31%)	4 (16%)	4 (17%)	3 (14%)	3 (13%)	
Data sovereignty (tribal nations)	4 (11%)	5 (14%)	3 (12%)	1 (4%)	2 (10%)	2 (9%)	

*Notes*: The denominator for each category varies because the number of exceptions requested in that category did not equal the total of 69 awardees. Also, within each request for exception, awardees could choose more than 1 category of reason for exception (eg, they could say “not applicable to study population” and “negative impact on community relationship”). Percentages are rounded to the nearest whole number.

## DISCUSSION

Most CDE research initiatives rely heavily on expert consensus for data element recommendations for future studies.^32^ Our process was unique in that we engaged a wide range of experts but also solicited direct feedback from multiple stakeholders in the community. This approach to CDE selection and refinement helped achieve the RADx-UP mission objectives and provided input directly from projects on the frontline. That said, we balanced the need for timely response with scientific integrity, while also building consensus and ensuring CDEs were codeveloped in a responsible and inclusive manner. Although there is significant room for improvement, our CDE process provided valuable insights into medically underserved populations rarely included in clinical trials.

We believe these community-guided CDEs could serve as the foundation of a patient-centered informatics architecture in modern clinical trials. We hope this approach will strengthen the long-term sustainability of academic-community partnerships formed through RADx-UP, and will help define standardized, reusable data repositories (enhanced by CDE formation) to facilitate future research in these populations. By inclusion of the NIH RADx-UP CDEs, researchers can generate linkable data, build better tools for understanding cultural factors, and examine intricate details of disease burden across various US populations. This CDE drafting process should be carefully critiqued, revised, and improved upon to further address deficits in data with an emphasis on building community trust and understanding local concerns.

In this project, the goal of RADx-UP was to engage historically marginalized populations in designing research variables and data collection strategies. Unfortunately, it is too early to say if these CDEs had the desired effect on data collection. However, in the context of a global pandemic, we successfully developed community-informed CDEs. In 2022, we will perform a detailed analysis of the CDEs used in data collection, which will likely serve as a measure of success for this work. An important part of the overall process was the reduction in number and refinement of the initial CDE list, improvements in the CDE language (based on project feedback), and the ability for most projects to successfully collect and transmit these CDEs. Based on anecdotal feedback from the projects and the general theme of comments during our project-wide meetings, we believe the Data Conformance dashboard and data status updates were successfully used by the projects to improve the data conformance, completeness, and validity of their CDEs. However, this has not been formally evaluated and is an area we will likely explore in the next phase of our data analysis.

As observed during RADx-UP, the community-engaged process can present a myriad of challenges. For example, some RADx-UP projects felt the CDE drafting process was rushed, insensitive, and may have inadequately considered community concerns. Based on the survey data from the 69 RADx-UP projects, many found the initial CDEs to be culturally incompatible, with the possibility of worsening stigmas among marginalized communities who may already perceive academia in an unfavorable light. As discussed above, there were persistent concerns over data privacy and CDE relevance. These concerns are significant, and key lessons learned from our CDE process include the importance of clear messaging, stating intent, assessing local factors, and providing more direct communications with stakeholders in the community. To improve the CDE selection and harmonization process, researchers must foster trust and mutual respect, balance expertise across communities, and overcome barriers through better understanding of the populations they serve.

Given the delicate nature of data sharing and lack of research engagement with underrepresented minorities, we highlighted the major survey feedback concerns over data protections and the potentially negative impacts on future community-informed research. Although exceptions to collecting NIH RADx-UP CDE categories provided some level of relief, careful drafting of CDE language was important to mitigate any negative or unintended impacts on participant and project relationships. Also, when collecting data like Social Security numbers and personal identifiers, it is understandable that there would be concerns over data privacy regardless of deidentification measures. With the prevalence of misinformation^33^ and the recent expansion of digital health in the United States,^34^ responsible privacy protections must be integral parts of future CDE drafting. As data collection and sharing initiatives have grown during the COVID-19 pandemic,^35^^,^^36^ and resulted in the creation of large health data repositories, privacy concerns will likely intensify.^37^^,^^38^

For example, data privacy tensions are exacerbated by the increasing use of wearables, personal health apps, and fertility trackers^39^ as well as the recent entry of large information technology (ie, “Big Tech”) companies into the clinical research industry.^40^ Currently, these entities collect and share massive personal “health-related” data with minimal regulatory oversight which can further misinformation and mistrust. More recently, Verily’s establishment of community COVID-19 testing—an online site to screen people for test eligibility—was met with harsh criticism from privacy advocates and resistance from communities concerned over exploitation.^41^ Google and Apple saw similar public outcry when they created a Bluetooth proximity data app for contact tracing,^42^ These examples highlight the delicate balance between data collection and data privacy, along with the potential negative impacts on clinical research. With a growing emphasis on standardizing data collection to facilitate discovery, interpretation, and reuse, privacy regulations and applicable policies should align with the public reality.

These data protection concerns present ongoing challenges that community-informed CDEs could help address. Such actions could mitigate adverse impacts on clinical research enrollment and foster public trust if drafted properly. With the volume of data privacy concerns reported by RADx-UP projects, such considerations are critical for future studies in underserved populations and should be considered by all clinical investigators using CDEs to enable more robust analysis. Well-crafted CDEs that are culturally sensitive and revised through a participatory process could help ensure research questions are relevant, easy to read, and nonintrusive on participants’ privacy. When CDEs are revised in this manner, the information obtained is kept to a minimum necessary and strikes a balance between data privacy and robust data analysis. This model and process could form the modern blueprint for all health disparities research across the United States.

## CONCLUSION

The RADx-UP consortium, together with NIH, balanced the need for rapid action during a pandemic with the goal of collecting meaningful, harmonized, and practical data. Our results show that a community-informed approach to CDE drafting can be rapidly scaled and implemented—overcoming language barriers, data privacy concerns, and technical challenges with data management, while also training projects through various formats fitting their local needs. Our experience highlights the challenges in implementing CDE data collection across many projects, including IRB approvals, data use agreements, data sharing, respecting data sovereignty rights of tribal nations, data submission and conformance methods, and quality improvement measures for identifying missing data. We also illustrate the power of data visualization tools using the CDE dashboard and its ability to facilitate hypothesis-driven research questions. We hope this work will become part of future roadmaps for clinical researchers working with underserved populations. Communities can benefit from having researchers select initial CDEs that are better informed and more aligned with the populations they serve. Although our work highlights the need to further refine and standardize data collection across different communities, these RADx-UP CDEs provide a unique foundation for community-engaged research projects to build upon. In conclusion, our work shows that an inclusive, community-informed approach can be used to develop CDEs in underserved populations. Such an approach can create better CDEs needed in future public health research during and beyond the COVID-19 pandemic.

## FUNDING

The RADx-UP CDCC is funded through National Institutes of Health (NIH) emergency cooperative agreement 1U24MD016258. Funding for the RADx-UP program is provided by the Paycheck Protection Program and Health Care Enhancement Act, 2020 and the American Rescue Plan Act of 2021 (NIH grant U24 MD016258). The content is solely the responsibility of the authors and does not necessarily represent the official views of the NIH.

## AUTHOR CONTRIBUTIONS

GAC, MC-W, EMD, KM, LMW, LJ, JT, AR, GC, and WAK conceived or designed the work, or acquired, analyzed, or interpreted data for the work; drafted the manuscript or revised it critically for important intellectual content; reviewed and approved the manuscript for submission; and agree to be accountable for all aspects of the work and ensure that questions of accuracy and integrity are investigated.

## SUPPLEMENTARY MATERIAL

Supplementary material is available at *Journal of the American Medical Informatics Association* online.

## Supplementary Material

ocac097_Supplementary_Data

## Data Availability

The CDE data will be available from the NIH RADx Data Hub.
