# Supplementary material for: Standardizing, harmonizing, and protecting data collection to broaden the impact of COVID-19 research: the rapid acceleration of diagnostics-underserved populations (RADx-UP) initiative
Source: J Am Med Inform Assoc. 2022 Jun 9;29(9):1480–8. doi: 10.1093/jamia/ocac097 (PMC9382379; doi:10.1093/jamia/ocac097)
Supplement: ocac097_Supplementary_Data [file ocac097_supplementary_data.zip › ocac097_Supplementary_Data/NIHCDETrackingForm_RADxUPCDCCD.pdf]

NIH CDE Tracking Form

RADx-UP Projects,

Please complete the RADx-UP NIH Common Data Elements (CDEs) Tracking Form, found below.

Each project must complete this form to provide information on data linkages even if you do not wish to request an exception.

Please reach out to your EIT with any questions.

Project number: [record_id]

Institution: [name]
Project name: [proj_spef_title]

EIT: [eit_num]
Project type: [proj_type]

This form will be completed by all RADx-UP projects and will be used to document and track the following information:

SSN Questions for all projects CDE Exception Requests and NIH Review Decisions of those Exception Requests Individual CDE exceptions for Tier 1 CDEs: This request is for a list of individual Tier 1 CDEs that you will not collect at all, with any of your project's participants. Participant Group exception Request: This request is for specific participant groups/arms for whom you do not believe it is feasible to collect CDEs. This section is only for requests related to CDEs that you will collect with some participants and not others within your Project. You do not have to re-address CDEs for which you already requested a Individual CDE Exceptions. CDE Wording Change Request: This request is for proposed wording changes to any Tier 1 and/or Tier 2 CDEs, including changes to CDE questions or answer choices. Changes to answer choices should include a description of how the changed answer choices map to the original CDE answer choices. Other Exception Requests: This request is for any other exception requests related to CDEs. At a minimum, all projects should answer the three questions related to SSN collection and data linkage in the next section.

Every project will complete this form before they start data collection. If your form includes an exception request, you will also complete a new form when you receive a response back from the NIH about your exception request and for any future requests and review decisions.

Are you completing this form as a New Request or in response to the NIH Review Decision?

New Request = First time CDE exception request submission OR a subsequent request with changes

NIH Review Decision = Updating the request form to reflect the decisions made following NIH's review

◯ New Request ◯ NIH Review Decision ◯ Study Closed Default (for CDCC internal use only)

Is the CDE Exemption valid to use for reporting? (CDCC Internal Use Only) ◯ Valid ◯ Not Valid

---

CDE Exemption Date - Autofilled when user first accesses ______________________________

SSN Questions - All Projects

You do not have to submit an exception request for Tier 2 identifiers if you are not collecting them, including social security number. However, SSN is needed for data linkages to external data sets such as insurance claims data or national death index data.

In order to plan for these external data sets, the CDCC needs to be able to estimate how many projects will either:

Provide SSN for the CDCC to use to obtain these external data sets Link external data sets to their own data and include this linkded data in their transfer to the CDCC   Please provide answers to the SSN questions for CDCC tracking.

Is your project collecting SSN?	◯ Yes<br>◯ No
Is your project sharing SSN with the RADx-UP CDCC?	◯ Yes<br>◯ No
What linkages will you be able to complete at your site prior to transferring data to the CDCC?	☐ Medicare claims<br>☐ Other insurance claims<br>☐ National Death Index<br>☐ Electronic Health Records<br>☐ Veteran's Affairs (VA) data<br>☐ None

Exception Requests

New Requests: Select which of the following CDE exceptions you are requesting.<br><br>NIH Review Decisions: Update your previously selected CDE exception to reflect the necessary changes to comply with your NIH review decision.<br><br>Select "No Exception Needed" if you are not requesting a CDE exception or none of your requested exceptions are allowed following the NIH review decision.	☐ Individual CDE Exception Request<br>☐ Participant Group Exception Request<br>☐ CDE Wording Exception Request<br>☐ Other CDE Exception Request<br>☐ No Exception Needed

Individual CDE exception Request for Tier 1 CDEs

New Requests: Select all Tier 1 CDEs for which you are requesting an exception.

NIH Review Decisions: Update previous selections as needed to conform to the outcome of your NIH review decision; modify all individual CDEs as necessary.

- ☐ Consent
- ☐ Location
- ☐ Sociodemographics
- ☐ Housing Employment and Insurance
- ☐ Work PPE and Distancing
- ☐ Medical History
- ☐ Health Status
- ☐ Vaccine Acceptance
- ☐ Testing
- ☐ COVID Test
- ☐ Symptoms
- ☐ Alcohol and Tobacco
- ☐ Identity (name, address, phone numbers, email, date of birth)

The exception selections will apply to all CDE versions - v1.0, v1.1, v1.2, and Pediatric.

Make selections based on variables' names, not field labels.

Consent CDEs

	Collecting and sharing with the CDCC	Collecting but not sharing with the CDCC	Not collecting
consent_given: Is consent required for this study?	○	○	○
consentdt_mdy: Date of Consent	○	○	○
consent_ident: I agree to let The Duke Clinical Research Institute to collect all identifiable information.	○	○	○
consent_ssn: I agree to let The Duke Clinical Research Institute to collect my Social Security number.	○	○	○
consent_zip: I agree to let The Duke Clinical Research Institute to collect only my zip code and no other identifiable information.	○	○	○
consent_recontact: I agree to be contacted for future research.	○	○	○

Location CDEs

	Collecting and sharing with the CDCC	Collecting but not sharing with the CDCC	Not collecting
current_county: County	○	○	○
zip_code: Zip Code	○	○	○

Sociodemographic CDEs

	Collecting and sharing with the CDCC	Collecting but not sharing with the CDCC	Not collecting
sociodem_date_mdy: Date of Sociodemographic Data Collection	◯	◯	◯
race_ethn_race: What is your race?	◯	◯	◯
race_ethn_asian_detail OR race_ethn_asian_detail_2:	◯	◯	◯
race_ethn_islander_detail OR race_ethn_islander_detail_2:	◯	◯	◯
race_ethn_orig_other: Specify other origin.	◯	◯	◯
race_ethn_hispanic: Are you of Hispanic, Latino, or Spanish origin?	◯	◯	◯
race_ethn_hispanic_detail OR race_ethn_hispanic_detail_2: Please specify your origin	◯	◯	◯
race_ethn_hispanic_other: Please specify other Hispanic, Latino, or Spanish origin.	◯	◯	◯
age_yrs: Age	◯	◯	◯
bio_sex_birth OR bio_sex_birth_2: What was your sex assigned at birth?	◯	◯	◯
gender_identity_term: What terms best express how you describe your gender identity?	◯	◯	◯
pregnancy_status: Are you currently pregnant?	◯	◯	◯
sex_orient_id: Which of the following best represents how you think of yourself at this time?	◯	◯	◯
edu_years_of_school: What is the highest level of education you have achieved outside or in the United States?	◯	◯	◯

Housing Employment And Insurance CDEs

	Collecting and sharing with the CDCC	Collecting but not sharing with the CDCC	Not collecting
housing_date_mdy: Date of Housing, Employment and Insurance Collection	○	○	○
household_famgen OR household_famgen_2: What best describes your family at home:	○	○	○
household_homeless: Are you currently living in transitional housing, staying in a shelter, or experiencing homelessness?	○	○	○
household_congregate OR household_congregate_2: Do you live in any of these?	○	○	○
household_other: Where do you stay/live?	○	○	○
jobloss_covid19: Have you, or has anyone in your household, experienced a loss of employment income since the start of the COVID-19 pandemic (March 2020)?	○	○	○
current_employment_status: We would like to know about what you do -- are you working now, looking for work, retired, keeping house, a student, or something else?	○	○	○
cur_employ_stat_specify: Current employment status, Other - specify	○	○	○
employed_ew: Are you considered an essential worker?	○	○	○
employed_healthcare: Would any of these describe where you work?	○	○	○
hi_coverage_type: What is the primary kind of health insurance or health care plan that you have now?	○	○	○

hi_loss_covid: Did you lose health coverage because of the COVID-19 pandemic?	◯	◯	◯
covid_pandemic_challenges_healthcare: Getting the health care I need (including for mental health)	◯	◯	◯
covid_pandemic_challenges_abode: Having a place to stay/live	◯	◯	◯
covid_pandemic_challenges_food: Getting enough food to eat	◯	◯	◯
covid_pandemic_challenges_water: Having clean water to drink	◯	◯	◯
covid_pandemic_challenges_medications: Getting the medicine I need	◯	◯	◯
covid_pandemic_challenges_transportation: Getting to where I need to go	◯	◯	◯
language_english: Do you speak a language other than English at home?	◯	◯	◯
language_spoken: What language(s)	◯	◯	◯
language_spoken_other: Specify other language(s)	◯	◯	◯
family_income: In 2019, what was your total household income before taxes?	◯	◯	◯

Work PPE and Distancing CDEs

	Collecting and sharing with the CDCC	Collecting but not sharing with the CDCC	Not collecting
work_ppe_date_mdy: Date of Work PPE and Distancing Collection	◯	◯	◯
work_wash: In your workplace, do you have access to necessary facilities to wash?	◯	◯	◯
work_closecont: Does your work require you to be in close contact (i.e. within 6 ft) with others?	◯	◯	◯
work_ppe: In your workplace, do you have access to necessary personal protective equipment (PPE)?	◯	◯	◯

Medical History CDEs

	Collecting and sharing with the CDCC	Collecting but not sharing with the CDCC	Not collecting
med_hx_date_mdy: Date of Medical History Collection			
cc_imm: Immunocompromised condition			
cc_autoimm: Autoimmune disease			
cc_hypertension: Hypertension (HTN, high blood pressure)			
cc_diabetes: Diabetes			
cc_chronickd: Chronic kidney disease (CKD)			
cc_cancer: Cancer diagnosis and/or treatment within the past 12 months			
cc_cvd: Cardiovascular disease (CVD or heart disease)			
cc_asthma: Asthma			
cc_copd: Chronic obstructive pulmonary disease (COPD)			
cc_clung: Other chronic lung disease			
cc_sickle: Sickle Cell Anemia			
cc_depression: Depression			
cc_asud: Alcohol or substance use disorder			
cc_intrav: Intravenous drug use			
cc_othermh: Other mental health disorder			
cc_otherchroniccond: Other chronic condition			

Health Status CDEs

	Collecting and sharing with the CDCC	Collecting but not sharing with the CDCC	Not collecting
hlthstat_date_mdy: Date of Health Status Collection			
self_reported_height_coded: How tall are you without shoes?			
self_reported_height_feet: Feet			
self_reported_height_inches: Inches			

self_reported_height_meters: Meters			
self_reported_height_centimeters: Centimeters			
self_reported_weight_units: Please choose the units you would like to use for weight			
self_reported_weight_kgs: How much do you weigh without clothes or shoes?			
self_reported_weight_lbs: How much do you weigh without clothes or shoes?			
self_reported_health_status_assessment: Would you say your health in general is excellent, very good, good, fair, or poor?			
self_reported_disability: Do you have a disability that interferes with your ability to carry out daily activities?			

Vaccine Acceptance CDEs

	Collecting and sharing with the CDCC	Collecting but not sharing with the CDCC	Not collecting
vacc_date_mdy: Date of Vaccine Acceptance Collection			
flu_vaccinehistind: Have you ever received a flu vaccination?			
flu_vaccine_season OR flu_vaccine_season_2: Have you received a flu vaccine this season (last 6 months)?			
covid_vaccine: Have you received a COVID-19 vaccine?			
vaccine_avail: How likely are you to get an approved COVID-19 vaccine when it becomes available?			
vaccine_reasons: Why would you get a COVID-19 vaccine?			
vaccine_concerns: Why would you NOT get a COVID-19 vaccine?			

Testing CDEs

	Collecting and sharing with the CDCC	Collecting but not sharing with the CDCC	Not collecting
test_date_mdy: Date of Testing Collection	○	○	○
isolate_maintain_job: If you were to test positive for COVID-19, would you be able to isolate without losing your job?	○	○	○
quarantine_maintain_job: If you would be exposed to someone with COVID-19, would you be able to quarantine without losing your job?	○	○	○
tested_for_covid: Have you ever been tested for COVID-19?	○	○	○
tested_positive_for_covid: Have you ever tested positive for COVID-19?	○	○	○
positivemonth_covidtest: What month did you first test positive for COVID-19?	○	○	○
positiveyear_covidtest OR positiveyear_covidtest_2: What year did you first test positive for COVID-19?	○	○	○
recentmonth_covidtest: What month did you have your most recent COVID-19 test?	○	○	○
recentyear_covidtest OR recentyear_covidtest_2: What year did you have your most recent COVID-19 test?	○	○	○
recentresult_covidtest: What was the result of your most recent COVID-19 test?	○	○	○
cov_tst_mthd: How were you tested for your most recent test?	○	○	○
test_accesswhere: I know where I can get COVID-19 testing in my community.	○	○	○
test_accesseasy: It is easy to get tested for COVID-19.	○	○	○

COVID Test CDEs

	Collecting and sharing with the CDCC	Collecting but not sharing with the CDCC	Not collecting
covid_test_date_mdy: Date of COVID Test Information Collection	◯	◯	◯
covid_test_target_disease_status OR covid_test_tar_dis_stat_2: Participant Testing Disease Status	◯	◯	◯
covid_test_approval: Quality and Regulatory	◯	◯	◯
covid_test_approval_other: Other approval	◯	◯	◯
covid_test_collection_setting: Test Collection Setting	◯	◯	◯
covid_test_collection_setting_other: Other setting	◯	◯	◯
covid_test_performed_location: Test Performed Location	◯	◯	◯
covid_test_performed_location_other: Other performed location	◯	◯	◯
covid_test_study_setting: Study Setting	◯	◯	◯
covid_test_study_setting_other: Other study setting	◯	◯	◯
covid_test_type: Test Method Target	◯	◯	◯
covid_test_type_other: Other method target	◯	◯	◯
covid_test_name: Test manufacturer (or LDT) and test name	◯	◯	◯
covid_test_specimen_type: Specimen Type	◯	◯	◯
covid_test_specimen_type_other: Other specimen type	◯	◯	◯
covid_test_specimen_collector: Specimen Collector	◯	◯	◯
covid_test_specimen_collector_other: Other specimen collector	◯	◯	◯
covid_test_collect_datetime: Date and time specimen collected	◯	◯	◯
covid_test_result_datetime: Date and time result received	◯	◯	◯

covid_test_result_sent_datetime: Date and time result sent to participant	○	○	○
covid_test_result_raw: Raw test result (if not a Positive/Negative/Failed report)	○	○	○
covid_test_result: Test result	○	○	○
covid_test_result_other: Other test result	○	○	○

Symptom CDEs

	Collecting and sharing with the CDCC	Collecting but not sharing with the CDCC	Not collecting
sym_date_mdy: Date of Symptom Collection	○	○	○
covid_fever: Fever or chills	○	○	○
covid_cough: Cough	○	○	○
covid_diffbreath: Shortness of breath or difficulty breathing	○	○	○
covid_fatique: Lack of energy or general tired feeling	○	○	○
covid_myalgia: Muscle or body aches	○	○	○
covid_headache: Headache	○	○	○
covid_olfactory: New loss of taste or smell	○	○	○
covid_runnynose: Sore throat, congestion or runny nose	○	○	○
covid_nausea: Feeling sick to your stomach or vomiting, diarrhea	○	○	○
covid_abpain: Abdominal Pain	○	○	○
covid_skinrash: Skin Rash	○	○	○
covid_other: Other	○	○	○

Alcohol and Tobacco CDEs

	Collecting and sharing with the CDCC	Collecting but not sharing with the CDCC	Not collecting
alcohol_date_mdy: Date of Alcohol/Tobacco Use Collection	○	○	○
lifetime_use_alcohol: In your entire life, have you had at least 1 drink of any kind of alcohol, not counting small tastes or sips?	○	○	○

alcohol_daysperweek: How often do you have a drink containing alcohol?	◯	◯	◯
smoker_cur_stat: Do you now smoke cigarettes?	◯	◯	◯
smoker_number: If you smoke every day, on average, how many cigarettes per day do you smoke?	◯	◯	◯
vaper_cur_stat: Do you now use electronic cigarettes every day, some days, rarely, or not at all?	◯	◯	◯

Identity CDEs

	Collecting and sharing with the CDCC	Collecting but not sharing with the CDCC	Not collecting
iden_date_mdy: Date of Identity Collection	◯	◯	◯
first_name: First Name	◯	◯	◯
last_name: Last Name	◯	◯	◯
current_street: Street Address	◯	◯	◯
current_street2: Street Address 2	◯	◯	◯
current_city: City	◯	◯	◯
current_state: State or Territory	◯	◯	◯
mobile_phone: Mobile Phone	◯	◯	◯
home_phone: Home Phone	◯	◯	◯
other_phone: Other Phone	◯	◯	◯
personal_email: Personal Email	◯	◯	◯
other_email: Other Email	◯	◯	◯
preferred_contact_method: Preferred Method of Contact	◯	◯	◯
dob_mdy: Date of Birth	◯	◯	◯

Tier 1 Exception Justification

Tier 1 CDE Exception Justification: For each variable you listed above, please provide a justification for not inlcuding the specific CDE in your study. If the same justification applies to multiple variables, please write the justification once with the list of variables.

Ex. Justification:

Study is being conducted with a vulnerable population for whom identity is highly protected due to stigma against the community and risk to community members related to being identified (e.g. transgender populations, people living with HIV/AIDS, sex workers, immigrant populations). Because of this, we will not be transferring identifiable information from our study to the RADx-UP CDCC. It would break trust with our community and make it difficult to reach recruitment goals. Our community advisory board has advised us not to transfer identifiable information. Therefore, we will not be submitting these CDEs: consent_ident, consent_ssn, consent_zip, consent_recontact or any CDEs in the identifiers section of the tier 1 CDEs.

____

If you would prefer to upload a document with your justification for your tier 1 exceptions, please do so here.

Tier 1 Identifiers

If you are not sharing tier 1 identifiers with the CDCC, is your project able to act as a patient re-contact registry by distributing information about upcoming studies to your patient pool?

- ◯ Yes
- ◯ No

If your site is not sharing location identifiers with the CDCC (address with zipcode), will your site be able to use identifiers at your site to link any of the following data types to your data prior to sending data to the RADx-UP CDCC:

- ☐ US Census Data / American Community Survey (ACS)
- ☐ Measures derived from the Census / ACS (e.g. Area Deprivation Index, Neighborhood Socioeconomic Status, Social Vulnerability Index)
- ☐ Water Pollution
- ☐ Air Pollution
- ☐ None

Participant Group exception Requests

Participant Group exception request: Please provide a detailed description of the participant groups for whom you are newly requesting the participant group exception. (Note: For NIH Review Decisions, please update or remove all previous descriptions to reflect the results of your review decision.)

Include whether you are requesting exception from collecting all CDEs with these groups or just a portion of the CDEs. If this exception is only for a portion of the variables, include a list of the specific variables.

You do not need to list any CDEs for which you requested individual CDE exceptions above.

____

Participant Group justification: Please provide a justification for requesting this participant group exception request. (Note: For NIH Review Decisions, please update or remove the justification to reflect justifications only for those exceptions approved by the NIH.)

Example justification:

50% of our participants will not be consented and only anonymous data will only be pulled from medical record review. For this 50% of our participant pool we will not submit any CDEs. For the other 50% of our participants who are consented we will collect surveys including CDEs and submit CDEs to the RADx-UP CDCC.

____

If you would prefer to upload a file containing your participant group exception justification, please do so here.

CDE Wording Change Request

CDE wording changes: Any CDEs for which you are requesting wording changes to the question stem or the response options, please use the appropriate template below to provide details of the proposed changes. (Note: For NIH Review Decisions, please provide an updated form that only includes wording change requests that were approved by the NIH.)

To request changes, please follow these steps:

Download the attached template below that matches the version of CDEs your project is using: New Phase II projects should use the "CDE1.4" file; all other projects use "CDE1.2" file.

Remove any CDEs for which you are not requesting wording changes.

To the CDEs for which you are requesting wording changes:

column D - complete for changes you wish to make for a question stem,

column F - complete for changes you wish to make to response options,

column G - complete to indicate how your updated response options correspond to the RADx-UP CDCC response

options.

Save your file.

Upload your file in the next field.

[Attachment: "CDE1.2_RADxUP_CDEWordingChangeTemplate_ExceptionRequest.xlsx"]

[Attachment: "CDE1.4_RADxUPPhase2_WordingChangeTemplate_ExceptionRequest.xlsx"]

Please upload the file with your requested CDE wording changes here. Please use the provided template above.

CDE Wording Change justification: Please provide a justification for each CDE wording change request listed above. (Note: For NIH Review Decisions, please update or remove the justification to reflect justifications only for those exceptions approved by the NIH.) ____________________

If the same justification applies to multiple variables, you may write the justification once with the list of variables.

If you would prefer to upload a document with your wording change justification, please do so here.

Other exception Requests

Other Exception Requests: Please indicate any additional exception requests from collecting or sharing RADx-UP CDEs. (Note: For NIH Review Decisions, please update or remove the information here to reflect the outcome of your NIH review.) ____________________

Other Exception Request justification: Please provide a justification for this exception request.(Note: For NIH Review Decisions, please update or remove the justification to reflect justifications only for those exceptions approved by the NIH.) ____________________

Email Confirmation

Date of exception request.

Click the "Now" button before submitting. ____________________

Enter the estimated date this NIH decision will be implemented in your data collection. ____________________

Add your email to receive the confirmation pdf ____________________
