# Supplementary material for: Standardizing, harmonizing, and protecting data collection to broaden the impact of COVID-19 research: the rapid acceleration of diagnostics-underserved populations (RADx-UP) initiative
Source: J Am Med Inform Assoc. 2022 Jun 9;29(9):1480–8. doi: 10.1093/jamia/ocac097 (PMC9382379; doi:10.1093/jamia/ocac097)

**Supplemental Figure 1.**

**CDCC Data-Conformance Screen Shot for RADx-UP Projects**

<https://app.powerbi.com/view?r=eyJrIjoiYjllOWQ0MTktMTJlNC00ZTUwLTkxODgtYzViNDE5MDYwOTMyIiwidCI6ImNiNzJjNTRlLTRhMzEtNGQ5ZS1iMTRhLTFlYTM2ZGZhYzk0YyIsImMiOjF9&pageName=ReportSection634a83264d3ae8207300>


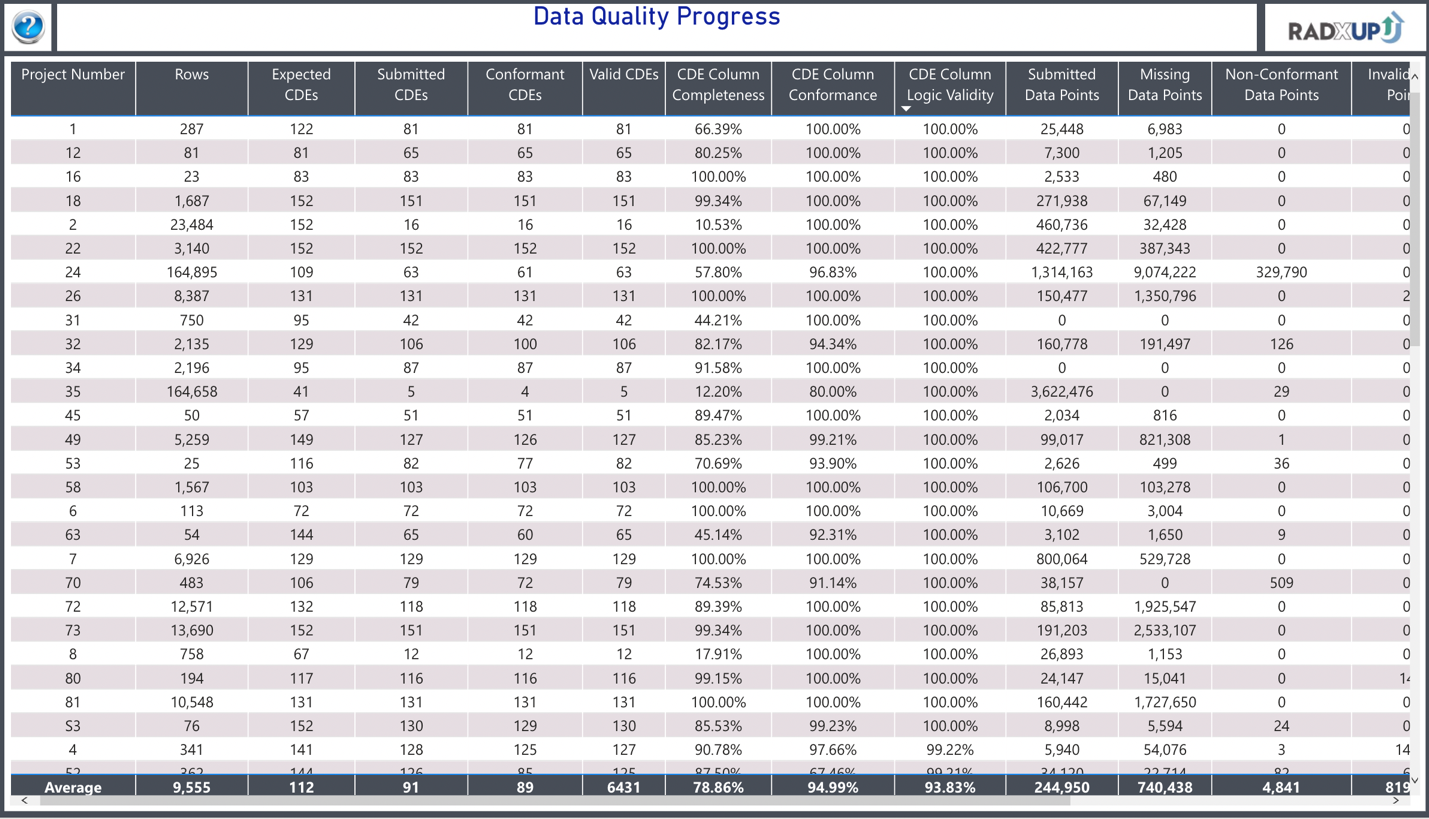

Supplement: ocac097_Supplementary_Data [file ocac097_supplementary_data.zip › ocac097_Supplementary_Data/RADX0003_Supplemental Figure 1_Data Conformance Dash.docx]
