# Supplementary material for: Standardizing, harmonizing, and protecting data collection to broaden the impact of COVID-19 research: the rapid acceleration of diagnostics-underserved populations (RADx-UP) initiative
Source: J Am Med Inform Assoc. 2022 Jun 9;29(9):1480–8. doi: 10.1093/jamia/ocac097 (PMC9382379; doi:10.1093/jamia/ocac097)
Supplement: ocac097_Supplementary_Data [file ocac097_supplementary_data.zip › ocac097_Supplementary_Data/RADX0003_Supplemental Material_CDEs Feedback Survey 2020.pdf]

# RADx-UP Common Data Elements - Feedback Form

Thank you in advance for your prompt, thorough review of the initial draft RADx-UP Common Data Elements (CDEs) to guide your data collection and research.

---

Will you be able to complete all of the RADx-UP Common Data Elements questions with your study participants?

◯ Yes
◯ No

---

Which category or categories contain questions you anticipate not being able to collect?
(select all that apply)

☐ Identity
☐ Consent
☐ Symptoms (COVID-19)
☐ Demographics
☐ Disability
☐ Employment
☐ Health Insurance
☐ Health Status
☐ Housing
☐ Medical History
☐ Medications
☐ Vaccine Hesitancy

---

Please explain your concerns or anticipated challenges to implementing those questions:

---

Do you have recommendations for alternative Common Data Elements questions within the existing categories?

◯ Yes
◯ No

---

What are the modules/question(s) for which you have recommendations?
(select all that apply)

☐ Identity
☐ Consent
☐ Symptoms (COVID-19)
☐ Demographics
☐ Disability
☐ Employment
☐ Health Insurance
☐ Health Status
☐ Housing
☐ Medical History
☐ Medications
☐ Vaccine Hesitancy

---

What is the alternative question? (1)

__________________________________

---

What is the source of this question?
[rad_cde_5_b_1]

__________________________________

Add another alternative question? ☐

What is the alternative question? (2)

________________________________

What is the source of this question?
[rad_cde_5_b_2]

________________________________

Add another alternative question? ☐

What is the alternative question? (3)

________________________________

What is the source of this question?
[rad_cde_5_b_3]

________________________________

Add another alternative question? ☐

What is the alternative question? (4)

________________________________

What is the source of this question?
[rad_cde_5_b_4]

________________________________

Add another alternative question? ☐

What is the alternative question? (5)

________________________________

What is the source of this question?
[rad_cde_5_b_5]

________________________________

Add another alternative question? ☐

What is the alternative question? (6)

________________________________

What is the source of this question?
[rad_cde_5_b_6]

__________________________________

Add another alternative question? ☐

What is the alternative question? (7)

__________________________________

What is the source of this question?
[rad_cde_5_b_7]

__________________________________

Add another alternative question? ☐

What is the alternative question? (8)

__________________________________

What is the source of this question?
[rad_cde_5_b_8]

__________________________________

Add another alternative question? ☐

What is the alternative question? (9)

__________________________________

What is the source of this question?
[rad_cde_5_b_9]

__________________________________

Add another alternative question? ☐

What is the alternative question? (10)

__________________________________

What is the source of this question?
[rad_cde_5_b_10]

__________________________________

Add another alternative question? ☐

What is the alternative question? (11)

__________________________________

What is the source of this question?
[rad_cde_5_b_11]

__________________________________

Add another alternative question? ☐

What is the alternative question? (12)

__________________________________

What is the source of this question?
[rad_cde_5_b_12]

__________________________________

Do you have recommendations for additional Common Data Elements questions?

◯ Yes
◯ No

What is the additional question? (1) __________________________________

What is the source of this question? __________________________________

Add another? ☐

What is the additional question? (2) __________________________________

What is the source of this question? __________________________________

Add another? ☐

What is the additional question? (3) __________________________________

What is the source of this question? __________________________________

Add another? ☐

What is the additional question? (4)

__________________________________

What is the source of this question?

__________________________________

The RADx-UP CDCC is planning to provide the final Common Data Elements in REDCap format, Qualtrics format, and as a PDF paper form. Which of these methods are you able to use to collect the Common Data Elements as part of your [research/protocol/study/activities]?

☐ REDCap
☐ Qualtrics
☐ Paper Form
☐ We will need another form of data collection

Does your institution's REDCap have "searching within a biomedical ontology" feature enabled so you can collect medications using RxNorm?

◯ Yes
◯ No
◯ Unsure

Explain:

What other feedback about the draft Common Data Elements would you like to share with the RADx-UP CDCC?
