# Supplementary material for: Standardizing, harmonizing, and protecting data collection to broaden the impact of COVID-19 research: the rapid acceleration of diagnostics-underserved populations (RADx-UP) initiative
Source: J Am Med Inform Assoc. 2022 Jun 9;29(9):1480–8. doi: 10.1093/jamia/ocac097 (PMC9382379; doi:10.1093/jamia/ocac097)
Supplement: ocac097_Supplementary_Data [file ocac097_supplementary_data.zip › ocac097_Supplementary_Data/RADX0003_Supplemental Material_Data Sharing ICF-template_v.2.4-18-22.docx]

# RADX-UP INFORMED CONSENT Data ShARING Template language

**Version 2.0: 02/17/2022**

**How to use this informed consent form template language:**

- This consent form is written at a 10^th^ grade reading level. Please edit your study’s Informed Consent Form to the appropriate educational level for your participant population.
- Text in **blue** indicates example text to include in your informed consent form.
- Text in *italicized black* are instructions and suggestions on what to include in certain sections or scenarios.
- Language from all sections below may be tailored to your study, but you are required to include language that covers:
  - The collection of all RADx-UP Tier 1 CDEs (as required by the terms and conditions of all RADx-UP awards) as well as additional Tier 2 CDEs as well as any other data your project is collecting
  - The transfer of collected data to the DCRI RADx-UP CDCC
  - The transfer of de-identified data from the DCRI RADx-UP CDCC to the NIH RADx Data Hub
  - The use of the de-identified data in the NIH RADx Data Hub by other researchers for future research (General Research Use)

# WHAT IS the NIH and RADx-UP?

The NIH stands for the National Institutes of Health. The NIH is part of the United States Department of Health and Human Services. The NIH’s purpose is to find new knowledge that will lead to better health for everyone. The NIH funded (provided support for) the RADx-UP program.

RADx-UP stands for Rapid Acceleration of Diagnostics - Underserved Populations. RADx-UP is a health research program to learn more about COVID-19 disease. If you join RADx-UP, we will gather some data (information) about you. We will combine these with data from other people who join RADx-UP. We will study the data from all who join to understand how to help more people at risk for or with COVID-19.

# WHAT WILL YOU ASK OF ME?

If you decide to join this study, we will gather data (information) about you. We will gather some of the data from you directly. We will gather some of the data from other places.

Examples of the information that we may collect from you or other places include, but not limited to: *[Tip: Being inclusive of all the information that will be collected will help avoid future IRB amendments as common data elements continue to be developed]:*

- We will ask you for basic information such as your name, date of birth, address, contact information, race, ethnicity, gender, language, health insurance status, disability, job, and household information including address history.
- We will ask you for your Social Security number (SSN), if you agree. We will use your SSN to help us link your data with other data, such as your electronic health record and Centers for Medicare and Medicaid Services, among others. We will not share your SSN with anyone or use it for any other purpose. You may choose not to provide your SSN and still be able to take part in this study.
- We will ask you information about COVID-19 that may include, but will not be limited to: information about any symptoms (a change in your health), test results, vaccination, and treatments. If you had a positive COVID-19 test, we will ask information about contact tracing (people who may have come in contact with you while you had COVID-19). We will ask about your medical history, including if you have or have not had vaccines and why, among others.
- We will ask you information about your health, education, family, home, relationships, and social life, among others.
- We may ask you to fill out questionnaires, surveys and other forms in order to collect the information above.

# What WILL YOU DO WITH MY DATA?

*Include this section only if you are using Colectiv for data collection:*

Data for this study will be collected using Colectiv, a web-based data collection system. Colectiv was developed for use by the RADx-UP program and is hosted at Duke University. This means that your research data will be hosted on data servers at Duke University. However, the space where your data is stored will only be accessible by 1) project staff at [insert institution name] who are IRB approved to work on this study and 2) Duke staff who need access to the system for system maintenance.

We will keep your data securely (which means with extra protection) within Colectiv. Some data [including/not including identifiers] from Colectiv will be transferred to the Duke Clinical Research Institute (DCRI) RADx-UP databases described below.

*Because the RADx-UP program will collect data for future research, you* ***must*** *include the following language. If you are not sharing direct identifiers with the CDCC, you may limit your description to the second database only:*

We will keep your data securely (which means with extra protection), along with the data from all the other people who take part in the RADx-UP program. Researchers will use the data to learn more about COVID-19 as well as other diseases and conditions.

The DCRI is a research group chosen by the National Institute of Health (NIH) to combine the data collected from everyone taking part in RADx-UP studies.

The DCRI will build two RADx-UP databases (systems that hold electronic information).

The first database will only hold information that can directly identify you. Examples are your name, address, email, and phone number.

- These data will be kept at the DCRI. The DCRI will not share these data with the NIH.
- If you agree to share your identifiable information by initialing below, your information will be linked with information from other sources, such as the Centers for Medicare and Medicaid Services and your electronic health record, among others.
- Only if you agree, by initialing below, the DCRI will keep information that can directly identify you and be used to contact you for future research studies. If you do not agree, this information will stay with your study team, as applicable.
- These data will stay in a password-protected secure electronic system and only staff responsible for maintaining the security of your data at the DCRI and IRB approved study staff will be able to see this information.

The second database will not hold information that can easily identify you. It will hold all the information you agree to give.

- You will be assigned a study code and you will only be identified in this database by this study code.
- It will not contain your name or other information that could easily identify you.
- We plan to transfer and keep these non-identifiable data in a secure database for COVID-19 research at the NIH. Other researchers may use these data for studies, other than the ones stated in this consent form.
- When using the data from this second database, researchers will not be able to link the data back to you.
- Because the data cannot be linked back to you, we will not contact you to inform you or ask your permission before sharing the data with researchers.

**Optional Questions:** *The following four questions are optional. Include them only if the questions are relevant to your project and you are providing an individual opt in/out option for specific variables and information to the participants:*

I agree to let the DCRI collect the following **identifiable** information: name, address, contact information, and dates collected as part of this study..

_____ Yes _____ No

Initials Initials

I agree to let the DCRI collect **my Social Security number**.

_____ Yes _____ No

Initials Initials

I agree to let the DCRI collect **only** **my zip code** and no other **identifiable** information.

_____ Yes _____ No

Initials Initials

I agree to be contacted for future research.

_____ Yes _____ No

Initials Initials

*Note: This section is required, but may be modified to be appropriate for privacy protections related to your entire study and include any language required by your institution.*

## HOW WILL YOU PROTECT MY PRIVACY?

Your privacy is **very** important to us. We will take great care to protect your privacy. However, there is always a chance that, even with our best efforts, your identity and/or information collected during this study may be accidentally released or seen by unauthorized persons. Here are a few steps we will take:

- Data will be [if using Colectiv add: collected via protected, secure applications and] stored on protected, secure computer systems. We will limit and keep track of who can see these data.
- Anyone who can see these data will have to use a password and multi-factor authentication.
- We will take steps to protect your information from others that should not be able to see it.
- When your data are shared with other researchers, they will not have information that can identify you.
- This project has a Certificate of Confidentiality from the United States government. Certificates of Confidentiality protect your privacy by blocking the release of identifiable, sensitive research information to anyone not connected to the research except when you agree, or in a few other specific situations.
