# Supplementary material for: Standardizing, harmonizing, and protecting data collection to broaden the impact of COVID-19 research: the rapid acceleration of diagnostics-underserved populations (RADx-UP) initiative
Source: J Am Med Inform Assoc. 2022 Jun 9;29(9):1480–8. doi: 10.1093/jamia/ocac097 (PMC9382379; doi:10.1093/jamia/ocac097)
Supplement: ocac097_Supplementary_Data [file ocac097_supplementary_data.zip › ocac097_Supplementary_Data/RADX0003_Supplemental Material_DTA_rev-4-18-2022.docx]

**AGREEMENT FOR DISCLOSURE AND TRANSFER OF CONFIDENTIAL INFORMATION AND PROTECTED HEALTH INFORMATION**

This **Agreement for Disclosure and Transfer of Confidential Information and Protected Health Information** (“**Agreement**”) is effective as of the date of the last signature below by and between ___________, located at ______ (“**Discloser**”) and Duke University, a tax-exempt research and educational institution located in Durham, North Carolina, acting for and on behalf of its Duke Clinical Research Institute (“**Duke**”).

**WHEREAS**, the National Institute On Minority Health And Health Disparities of the National Institutes of Health (“**Awarding Agency**”), has engaged Duke to serve as the Coordination and Data Collection Center (“**CDCC**”) for a federally-funded program of clinical research involving human subjects entitled: “*Rapid Acceleration of Diagnostics for Underserved Populations Coordination and Data Collection Center”* also referred to as “**RADx-UP CDCC**” (FAIN: U24MD016258, CFDA #93.310) (Duke IRB # Pro00106873) (the “**Project**”) under the direction of Duke’s investigator, Michael Cohen-Wolkowiez, MD (“**Duke Investigator**”);

**WHEREAS**, the Project aims to provide overarching support and guidance to the Awarding Agency on its administrative operations and logistics, facilitating effective use of COVID-19 testing technologies, supporting community and health system engagement and providing overall infrastructure for data collection, integration and sharing for Awarding Agency’s *Rapid Acceleration of Diagnostics for Underserved Populations* (“**RADx-UP**”) program;

**WHEREAS**, Discloser, under the direction of its lead investigator, [Project PI name] (“**Discloser Investigator**”), has received an award from the Awarding Agency to participate in the RADx-UP program and, accordingly, has agreed to share data with the Project;

**WHEREAS**, RADx-UP program includes the formation of a consortium (“**RADx-UP Consortium**”) to facilitate cooperation among various entities participating in RADx-UP program; Duke as the CDCC and Discloser are members of the RADx-UP Consortium;

**WHEREAS**, Duke has assisted the Awarding Agency to develop minimum necessary common data elements to guide RADx-UP program participants to submit research subjects’ data in support of the RADx-UP program (“**RADx-UP Common Data Elements**”);

**WHEREAS**, Discloser wishes to share with Duke, or Duke will be given access to (i) research subjects’ information that constitutes Protected Health Information (“**PHI**”), as defined in the Health Insurance Portability and Accountability Act of 1996, as amended (“**HIPAA**”), including, but not limited to, research subjects’ contact information and (ii) other information that Discloser considers to be confidential (“**Discloser Confidential Information**”) to enable Duke to: (a) obtain research subjects’ written informed consent/HIPAA authorization (“**Subject Authorization**”), RADx-UP Common Data Elements, related questionnaires, surveys and forms for performing data analyses and for collecting follow up data from research subjects; (b) better understand COVID-19 testing patterns among underserved and vulnerable populations; (c) strengthen the understanding of the impact of relevant data on disparities in infection rates, disease progression, and outcomes; (d) develop strategies to reduce disparities in COVID-19 testing; and (e) fulfill Duke’s obligation as the CDCC under the Project to provide de-identified Project data and the results of its analyses to the Awarding Agency (collectively, “**Duke Purpose**”); and

**WHEREAS**, Duke will share with Discloser, or Discloser will have access to, reports and resources in the Project’s RADx-UP resource library, confidential testing-related documentation, and other information that Duke considers to be confidential (“**Duke Confidential Information**”) to inform Discloser’s decisions and response to the COVID-19 crisis (“**Discloser Purpose**”). PHI, Discloser Confidential Information and Duke Confidential Information shall be collectively referred to hereinafter as “**Information**”.

**NOW, THEREFORE**, the parties hereby agree to the following terms and conditions:

1. Each party agrees that it shall treat Information it receives from the other party in strict confidence and shall avoid disclosure of the Information to any other person, firm or corporation, other than those who have a need to know the Information in order to carry out each party’s respective Purpose, and who are subject to confidentiality obligations sufficient to carry out the intent of this Agreement.
2. Discloser certifies that (i) PHI was collected with the Subject Authorization of the individuals to whom it relates, that such Subject Authorizations do not prevent the use of PHI for the Duke Purpose set forth herein, and that release of PHI for the Duke Purpose shall be approved by Discloser’s Institutional Review Board (“**IRB**”), or (ii) a waiver or alteration of consent and HIPAA authorization has been granted by Discloser’s IRB. Each party is responsible for obtaining and complying with any review or approval required by the respective party’s IRB or organizational policies.
3. Neither party shall transfer or sell the Information it receives to third parties or, as it relates to Duke, attempt to identify or contact any research subjects to whom the PHI pertains, except as necessary to fulfill each party’s respective Purpose, and as authorized by the relevant party’s IRB or by the Subject Authorization.

1. Excluding PHI, for which the following exceptions do not apply, neither party shall have any obligation, with respect to the other party’s Information or any part thereof that:
   1. is already known at the time of the disclosure from a source not associated with the Project or the RADx-UP program;
   2. becomes publicly known without the wrongful act or breach of this Agreement by a party;
   3. is rightfully received by the receiving party from a third party not associated with the Project or the RADx-UP program on a nonconfidential basis;
   4. is approved for release by written authorization of the disclosing party;
   5. is independently developed by a party without use of or reliance upon the other party’s Confidential Information; or
   6. is required to be disclosed by law or a court order, subject to the protections provided by the Certificate of Confidentiality provided by the Awarding Agency to all RADx-UP Consortium members.
2. Except as expressly provided in Section 1, Duke shall keep all PHI described in this Agreement in strict confidence. Each party’s obligations of confidentiality with respect to the other party’s Confidential Information (not PHI) shall continue for a period of five (5) years after the first to occur of termination of this Agreement or until the party returns or destroys the other party’s Confidential Information pursuant to Section 10 below. PHI shall be kept in strict confidence without limitation of time.
3. All records containing PHI shall be maintained under appropriate electronic and physical security. Discloser shall encrypt all PHI in transit. All transmission of PHI shall be through a secure web portal. Duke shall report to Discloser any use or disclosure of PHI not provided for in this Agreement of which Duke becomes aware, and Duke shall take reasonable steps to limit any further such use or disclosure.
4. Discloser shall adopt, implement, and maintain appropriate physical and electronic security controls to protect against unauthorized access of any PHI or personally identifiable information of research subject(s), or any Duke employees, agents or customers. Each party accepts responsibility and liability for its unauthorized disclosure of the other party’s Information, shall immediately notify the other party of any breach of data security or other unapproved release of the other party’s Information or other personally identifiable information, and shall take appropriate steps to limit any further such use or disclosure.
5. Each party shall use the other party’s Information solely for the receiving party’s respective Purpose and shall not be entitled to make any other use of the other party’s Information. No license or additional rights are provided to either party under any patent applications, copyrights, trade secrets, or other proprietary rights of the other party, and all Information provided to a party hereunder shall remain the property of the disclosing party.
6. The parties agree that neither shall receive any remuneration or compensation from other for the provision of Information to the receiving party under this Agreement, and as such, neither party’s Financial Conflict of Interest policy shall apply.
7. Upon completion of each party’s Purpose, the relevant party shall return all Information received from the disclosing party to that party, or at the disclosing party’s election, destroy all Information and provide the disclosing party with written certification of such destruction. Notwithstanding the foregoing, Duke shall be permitted to provide de-identified Project data and the results of Duke’s analyses to the Awarding Agency in order to fulfill the Duke Purpose and pursuant to the obligations of the RADx-UP Consortium. This Agreement shall terminate upon the return or destruction of all Information received from the other party; provided, however, that the obligations of confidentiality set forth herein shall continue as set forth in Section 5 above. Each party may retain one copy of the other party’s Confidential Information (not PHI) for the purpose of monitoring its obligations of confidentiality hereunder.
8. Should Duke commit a material breach of this Agreement with respect to PHI, which is not cured within thirty (30) days after Duke receives notice of such breach from Discloser, then Discloser will discontinue disclosure of PHI and will report the breach to the Secretary, Department of Health and Human Services.
9. This Agreement may not be assigned by either party without the prior written consent of the other.
10. This Agreement represents the entire understanding between the parties, and supersedes all other agreements, express or implied, between the parties as to its subject matter. Any alteration, modification, or amendment to this Agreement must be in writing and signed by authorized representatives of each party.

**IN WITNESS WHEREOF**, the parties have signed or caused this Agreement to be signed as of the dates indicated below.

**DUKE UNIVERSITY** [Institution Name]

By: By:

Susan E. Hayden, J.D. Name:

Director, Research Program Collaborations Title:

Office of Research Contracts

Date signed: Date signed:
